# Peri-implant bone resorption risk of anterior maxilla narrow single implants: a finite-element analysis

**DOI:** 10.1080/26415275.2022.2135516

**Published:** 2022-10-25

**Authors:** Ivan Onone Gialain, Leonardo Folmer Rodrigues da Silva, Marlene Kasumi Gantier Takano, Rafael Yagüe Ballester, Marina Guimarães Roscoe, Josete Barbosa Cruz Meira

**Affiliations:** a School of Dentistry, Department of Biomaterials and Oral Biology, University of São Paulo, São Paulo, Brazil; b “Finite element analysis in Dentistry” Course, School of Dentistry, Department of Biomaterials and Oral Biology, University of São Paulo, São Paulo, Brazil

**Keywords:** Crestal bone loss, strain energy density, anterior maxilla, narrow dental implants

## Abstract

**Statement of the problem:** Narrow implants have been recommended in high esthetic demand regions to ensure greater buccal bone thickness (BBT) and minimize soft-tissue recession due to insufficient bone support. However, a limited area of bone-implant interface can increase the risk of peri-implant bone resorption due to occlusal forces.

**Purpose:** This article encourages the use of evidence-based finite element analysis to optimize the aesthetic outcomes in maxillary lateral incisor single-supported implant crown by accurate biomechanical planning. This study aimed to analyze the best implant dimensions that would preserve the maximum BBT and avoid peri-implant bone resorption due to occlusal forces.

**Materials and methods:** A maxilla segment was constructed based on anthropological measurements. Four implant diameters (Ø = 3.25; 3.50; 3.75 or 4.00 mm) and two lengths (*L* = 10 or 13 mm) were simulated. The occlusal force parameters were defined to simulate clinical conditions. The bone resorption risk analysis was based on Frost’s mechanostat theory altering the strain output to strain energy density (SED). The peri-implant bone resorption risk indexes (PIBR_ri_) were calculated by dividing the average of the top ten SED elements of the cortical and trabecular buccal wall by the pathologic resorption limit for each bone.

**Results:** For trabecular bone, only the model Ø4.00_L13_ exhibited a low PIBR_ri_. For cortical bone, all models presented a low PIBR_ri_, except for models Ø3.25.

**Conclusion: **The selection of a 3.25 mm dental implant to preserve a 2 mm BBT should be avoided since it generates a high peri-implant bone resorption risk induced by occlusal overload.

## Introduction

Dental implants have become the most promising and advantageous treatment option for tooth loss rehabilitation. Despite the high survival rates of implant-supported single crowns [1], the aesthetic outcome of anterior maxilla treatments is not always predictable [2]. The maxillary anterior region is an aesthetically high-demanding zone, where a minor failure can jeopardize patients’ expectations and approval [3,4].

Soft tissue recessions are considered the most common aesthetic complications in single implant restorations and are often associated with a thin tissue biotype, insufficient bone volume during implant installation, or crestal bone resorption after implant function. Soft tissue [3,5] and bone [6–8] augmentation have been recommended before or during implant installation in unfavorable anatomy scenarios. Still, esthetic failures can also occur in a favorable anatomy scenario due to improper biomechanical or surgical planning, which can result in bone resorption.

Clinical guidelines of the ideal three-dimensional implant position usually highlight the importance of a sufficient buccal bone volume [9]. An adequate buccal bone thickness has been advocated to ensure proper soft-tissue support, avoid buccal bone resorption due to occlusal overload, and minimize the risk for peri-implant soft-tissue recessions [5,10,11]. Still, the critical buccal bone thickness (BBT) threshold is controversial [9]. A minimal BBT value of 2 mm [12], 1.5 mm [7], and 1 mm [11] have been reported. To follow the minimal 2 mm BBT, a narrow implant should be used for most maxillary lateral incisor implant-supported crowns even in a favorable scenario of alveolar process dimensions.

The use of narrow implants has been recommended to avoid augmentation need [13]. Still, from a biomechanical point of view, decreasing the implant diameter contributes to an increase in the risk for peri-implant bone resorption due to overload, since the stress and strain levels in the peri-implant bone are related to the bone-implant contact area [14]. When the mechanical stimulus in the peri-implant bone surpasses the pathological resorption threshold, the bone remodeling equilibrium is broken and an irreversible bone loss can occur [15].

Finite element analysis (FEA) is a powerful tool to evaluate the pathological bone resorption risk at the planning stage, preventing clinical failure. In recent years, there have been great advancements in bone remodeling simulation. Yet, most FE studies in Dentistry do not consider the bone remodeling theories, nor the mechanical stimulus limit that drives the cortical or trabecular bone to the pathological resorption window. Therefore, only qualitative comparisons can be made, usually confirming that the higher the implant diameter or length, the lower the stress and strain levels in peri-implant bone [16]. This FEA approach is limited to guiding the dentist in clinical decision-making since the bone stress and strain values are not converted into actual bone resorption risk index.

This study aimed to evaluate, by finite element analysis, the peri-implant bone resorption risk index (PIBR_ri_) of different implant diameters and lengths of implant-supported maxillary lateral incisor crown. The bone pathological resorption threshold was based on available scientific knowledge of bone remodeling induced by mechanical stimulus. The idea is that the calculated PIBR_ri_ will enable the dentist to incorporate the best available evidence of bone remodeling science into clinical decision-making. In addition, this article discusses the implications of ignoring the difference in material properties between cortical and trabecular bones when interpreting FEA outputs associated with bone resorption induced by mechanical overload.

## Materials and methods

**Figure 1.**
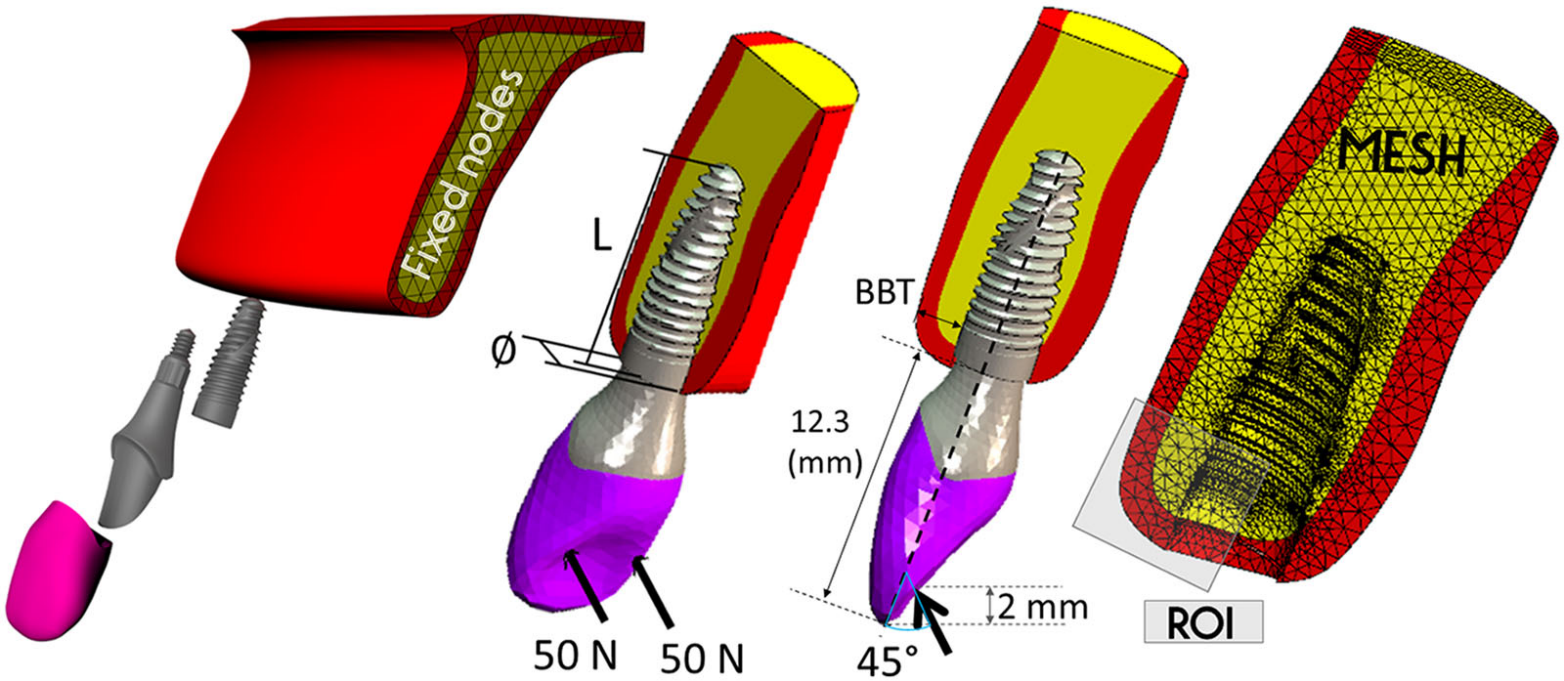
Finite-element model descriptions: geometry (mm), mesh, boundary conditions (fixed nodes), and occlusal loading (N: newton). L: implant length, ∅: implant diameter, BBT: buccal bone thickness, ROI: region of interest (only the mesial ROI is visible in the image, but distal ROI was also used to calculate the peri-implant bone resorption risk indexes).

Eight models of implant supporting lateral incisor crowns in the anterior maxilla were constructed (Figure 1) using Rhino3D software (version 7, Robert McNeel & Associates, Seattle, WA). MSC.Apex and MSC.Marc programs (MSC Software, Santa Ana, CA, USA) were used in the following steps of the finite element analysis. The implant geometries were modeled based on a NeoPoros dental implant (NeoDent, Curitiba, Brazil), combining four different diameters and two lengths (Table 1). The cone-morse abutment (model 114.753, NeoDent, Curitiba, Brazil) and the full-ceramic crown (imported from Brenes tooth library) were adjusted to the implant diameter, maintaining the same position and inclination for all models, but different buccal bone thickness (Figure 1 and Table 1).

**Table 1.** Simulated dental implant geometries.

Model	Implant diameter (mm)	Implant Length (mm)	Bone-implant interface area (mm^2^)	Remained buccal bone thickness (mm)
Ø3.25_L10_	3.25	10	115.45	2.000
Ø3.25_L13_	3.25	13	159.21	
Ø3.50_L10_	3.50	10	127.73	1.875
Ø3.50_L13_	3.50	13	176.52	
Ø3.75_L10_	3.75	10	140.54	1.750
Ø3.75_L13_	3.75	13	194.60	
Ø4.00_L10_	4.00	10	153.88	1.625
Ø4.00_L13_	4.00	13	213.47	

The host bone represented a maxillary D3 bone (type III), with a 1 mm cortical layer surrounding the trabecular bone. After a mesh convergence test, the four-node tetrahedral elements had a size from 0.30 mm up to 1.50 mm, with greater element refinement close to the bone-implant interface. The total number of elements for each simulation was between 241,532 and 344,218. All materials were considered isotropic, homogeneous, and linear (Table 2).

**Table 2.** Materials properties.

Material	Elastic modulus (GPa)	Poisson coefficient
Cortical bone	13.7	0.3
Trabecular bone	1.37	0.3
Dental implant (titanium)	113	0.3
Abutment	113	0.3
Lithium disilicate (crown)	95	0.3

Two occlusal loads of 50 N magnitude were applied respectively on the mesial and distal edges, at 45° to the implant long axis (Figure 1), simulating a total 100 N bite force with a 2 mm overbite [17–19]. A 100% osseointegration situation was assumed for all models. Full kinematic constraints were applied to the lateral planes of the maxillary bone.

**Figure 2.**
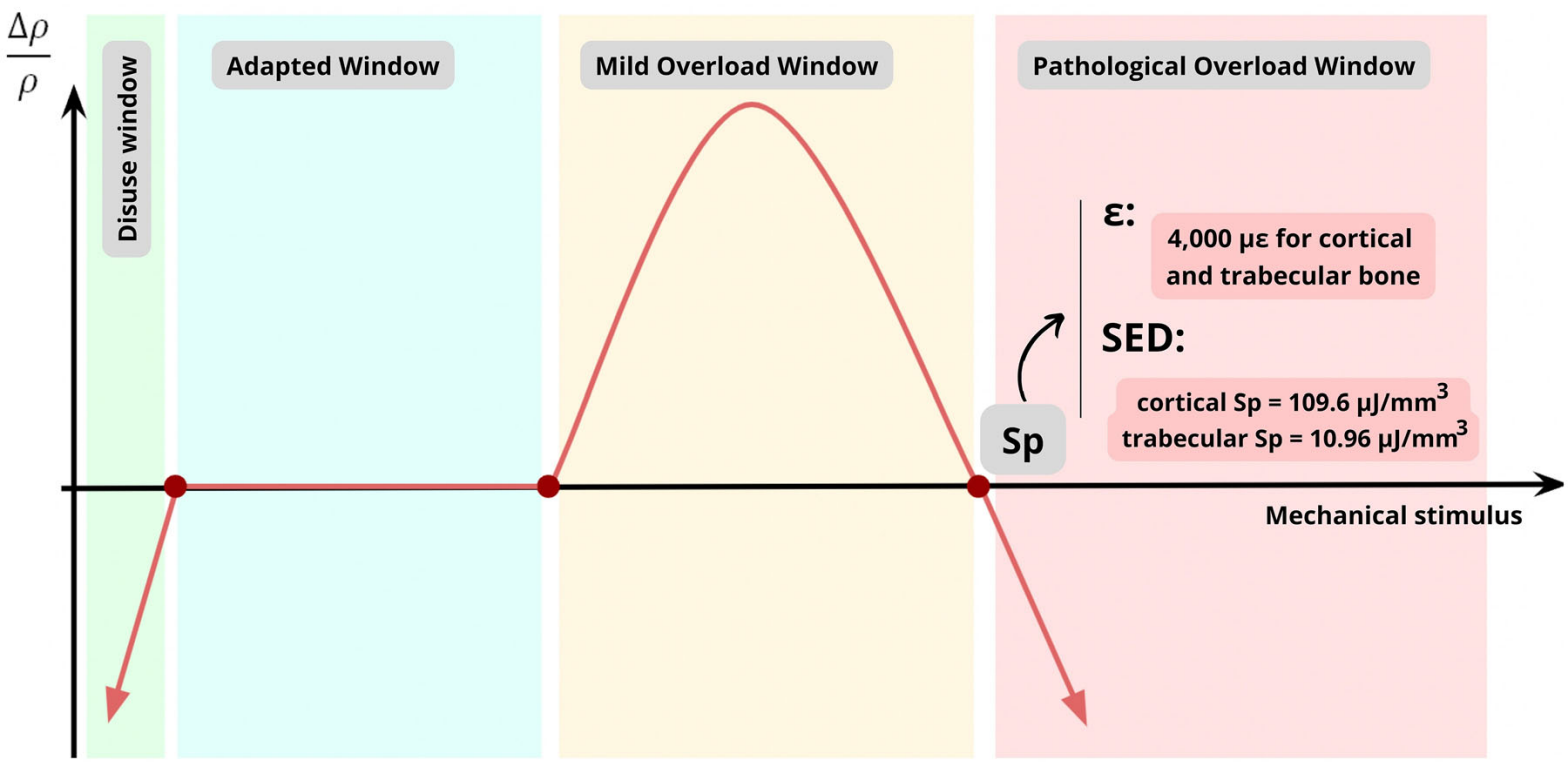
Frost’s mechanostat windows based on the bone density change $\;(\frac{\Delta \rho }{\rho })$ as a function of the mechanical stimulus. Sp: mechanical stimulus that drives the bone into pathologic resorption window. SED: strain energy density (in µJ/mm^3^). ε: strain (in µstrain = 1 µm/m). ρ: bone density. Δρ: bone density variation.

The peri-implant bone resorption risk analysis was based on Frost’s mechanostat theory (Figure 2) altering the strain output by strain energy density (SED). The SED pathological threshold (Sp) for cortical and trabecular bone was calculated according to the equations below:

(1) $$\mathrm{SED}\;=\;\frac{\sigma \varepsilon }{2}$$

(2) σ=Eε

(3) $${Sp}_{c}=\frac{E_{c}{{(\varepsilon }_{p})}^{2}}{2}$$

(4) $${Sp}_{t}=\frac{E_{t}{{(\varepsilon }_{p})}^{2}}{2}$$

where σ is the stress, ε is the strain, E is the elastic modulus,${\;\varepsilon }_{p}$ is the bone strain threshold to pathologic window (which is 4,000 µstrain according to Frost’s Mechanostat [15]), $E_{c}$ is the cortical elastic modulus (which was set as 13,700 MPa), $E_{t}$ is the trabecular elastic modulus (which was set as 1,370 MPa), ${Sp}_{c}$ is the SED pathologic threshold for cortical bone, and ${Sp}_{t}$ is the SED pathologic threshold for trabecular bone. By replacing ${\;\varepsilon }_{p},\;E_{c}\;$ and $E_{t}\;$ in Equations (3) and (4) by their corresponded values, the calculated Sp values were 109.6 µJ/mm^3^ for cortical bone (${Sp}_{c}$) and 10.96 µJ/mm^3^ for trabecular bone (${Sp}_{t}$).

The peri-implant bone resorption risk indexes (PIBR_ri_) were calculated by dividing the maximum SED values for peri-implant cortical and trabecular buccal wall by the correspondent SED pathologic threshold for each bone. Since element distortion can exaggerate calculated stresses, which may potentially cause inaccurate spike values, the maximum value of the SED was determined as the average of the highest 10 element values of the region of interest (ROI – buccal bone).

(5) $$\mathrm{cortical}\;{\text{PIBR}}_{\text{ri}}=\frac{\mathrm{SED}\;\mathrm{average}\;of\;\mathrm{top}\;10\;\mathrm{buccal}\;\mathrm{cortical}\;\mathrm{elements}}{{Sp}_{c}}$$

(6) $$\mathrm{trabecular}\;{\text{PIBR}}_{\text{ri}}=\frac{\mathrm{SED}\;\mathrm{average}\;of\;\mathrm{top}\;10\;\mathrm{buccal}\;\mathrm{trabecular}\;\mathrm{elements}}{{Sp}_{t}}$$

The indexes were divided into three categories:

- low resorption risk (PIBR_ri_ < 0.8),
- medium resorption risk (0.8 ≤ PIBR_ri_ ≤ 1.0),
- high resorption risk (PIBR_ri_ > 1.0).

## Results

### SED distribution

**Figure 3.**
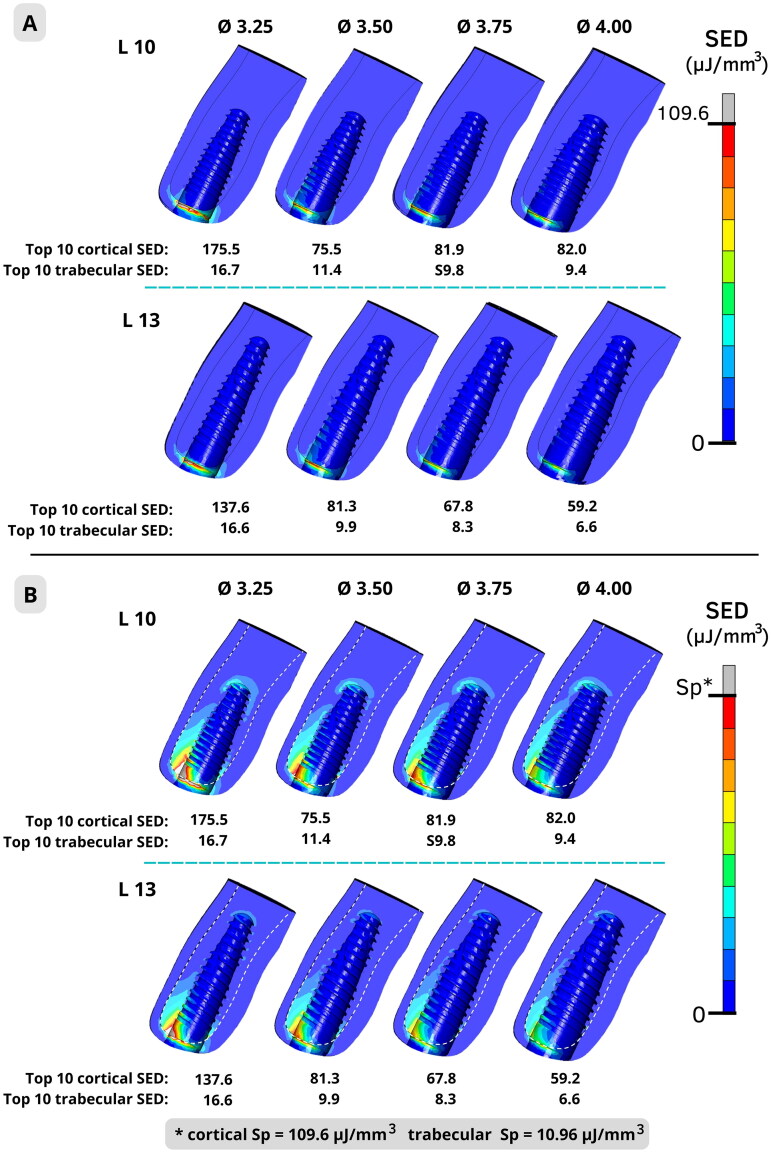
Strain energy density (SED) distribution and mean of top 10 SED for cortical and trabecular buccal bone. A: using the same color range adjustment for cortical and trabecular bone. B: using specific color range adjustments for cortical and trabecular bone, according to each bone SED pathologic resorption limit (Sp) value (109.6 µJ/mm^3^ for cortical and 10.96 µJ/mm^3^ for trabecular bone).

Figure 3(A) shows the peri-implant SED distribution, using the same range for cortical and trabecular bones. The scale was adjusted to present in gray the regions with SED ≥109.6 µJ/mm^3^ (${Sp}_{c}$). Figure 3(B) shows the same peri-implant SED distribution but considers different ranges for cortical and trabecular bones: the scale of trabecular bone was adjusted to present in gray the regions with SED ≥10.96 µJ/mm^3^ (${Sp}_{t}$), while the scale of cortical bone maintained the same scale as Figure 3(A) (${Sp}_{c}$).

By using specific scales, a more accurate interpretation of trabecular peri-implant bone resorption risk was achieved. The highest SED values can be seen at the cortical-trabecular interface, close to the implant shoulder level. For models Ø3.25, an evident gray region was observed for both cortical and trabecular layers and both implant lengths. For models Ø3.50 and Ø3.75_L10_, an almost unnoticeable gray region was observed only for trabecular bone. For models Ø3.75_L13_ and Ø4.00, no gray region was observed.

### Peri-implant bone resorption risk index (PIBR_ri_)

**Figure 4.**
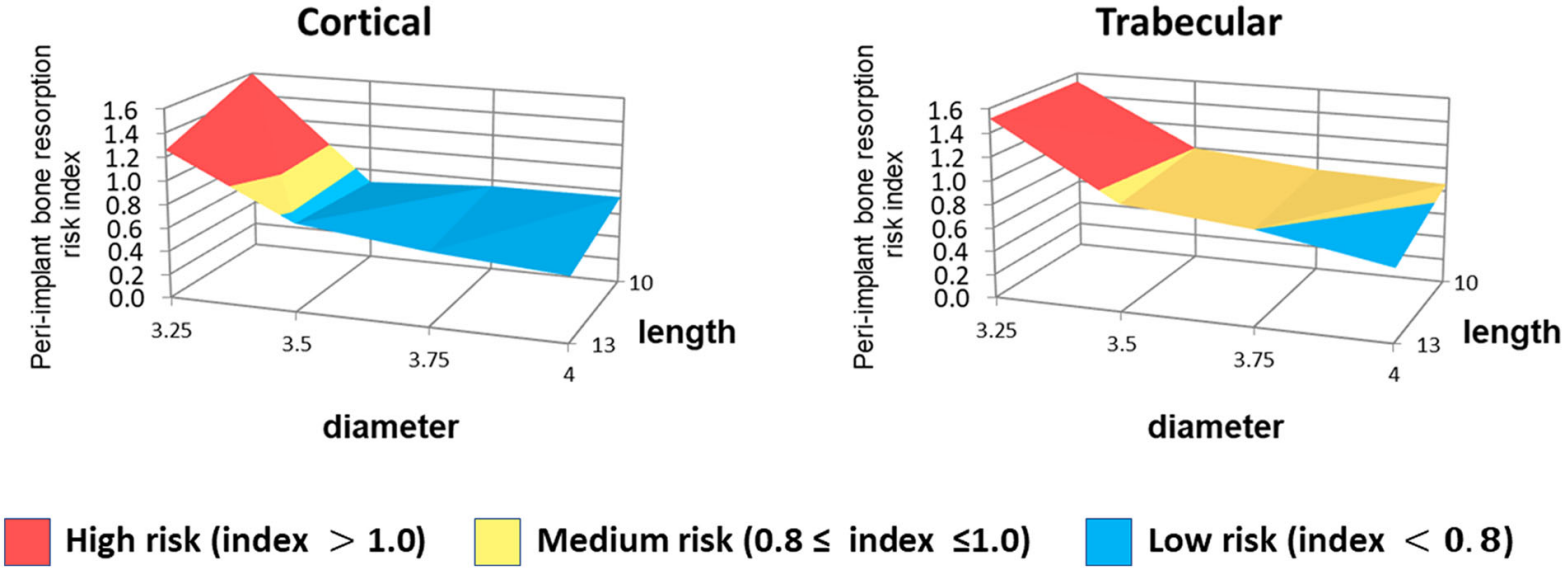
Peri-implant bone resorption risk index (PIBR_ri_) for cortical and trabecular bones.

Figure 4 presents the peri-implant bone resorption risk for cortical and trabecular bones. For cortical bone, high resorption risk indexes were observed for models Ø3.25, while low-risk indexes were observed for the other models. The trabecular bone indexes of models Ø3.50, Ø3.75, and Ø4.00_L10_ differed from the correspondent cortical ones, presenting a medium risk of resorption. Therefore, only Ø4.00_L13_ presented a low-risk index for trabecular bone.

## Discussion

The idea that bone shape is related to mechanical loading has been known since Galileo [20], but it was in the 1980s that the Mechanostat theory was established [21]. In the last decades, Frost’s mechanostat has been used to evaluate the change in bone density due to mechanical stimulus, in which the bone density is maintained by a biomechanical feedback system [22].

As specified by Frost [15,21,23], the bone response to a mechanical stimulus can be divided into four windows (Figure 2). In the adapted window, the bone remodeling is in equilibrium, therefore, no density change is observed. In the mild overload window, the osteogenic process is intensified to increase bone stiffness and, consequently, adapt to the overload condition. However, if the mechanical stimulus surpasses a threshold value, the bone is taken to the pathological overload window, in which an irreversible bone resorption process takes place. Therefore, a successful biomechanical planning of an implant-supported single crown must guarantee that the mechanical stimulus at the peri-implant bone stays below the pathological overload threshold (Sp) of the cortical and trabecular bones.

The determination of the bone Sp value is critical. According to Frost [15], when the strain in the bone is below 2,000 µstrain, the remodeling mechanism can easily repair the microdamage generated by cyclic mechanical load. Yet, when the strain exceeds 4,000 µstrain, microdamage overcomes the bone repair capacity, resulting in pathological overload resorption. Therefore, the 4,000 µstrain has been used as a reference for the Sp value for both cortical and trabecular bones [24,25].

The peri-implant bone strain (or stress) distribution can be easily obtained by finite element analysis. However, the strain (or stress) is a second-rank tensor quantity, which directional property has to be defined. It means that in finite element analysis, the output cannot be just ‘strain’ or ‘stress’: a specific component of the tensor has to be selected. Therefore, the mechanostat is theoretically meaningful, but the use of a strain value as a pathological overload threshold in FE studies remains questionable unless the exact component of the strain can be specified. To overcome this issue, Frost’s strain threshold has been converted into a strain energy density value (Equation (1)) in peri-implant bone remodeling simulations [7,22,26,27]. The advantage of SED is that it is a scalar quantity that measures the overall distortion energy; therefore, there is no need to determine the directional properties of the strain or stress tensor [22,28,29].

The SED value that drives the bone into the pathological overload window differs between cortical and trabecular bones (Equations (1) to (4)), since their elastic modulus are different ($E_{c}\neq E_{t}$) [28]. In the present study, the cortical elastic modulus ($E_{c}=$13.7 GPa) was ten times the value of trabecular bone ($E_{t}=$1.37 GPa), resulting in a ${Sp}_{c}$ (109.6 µJ/mm^3^) ten times higher than the ${Sp}_{t}$ (10.96 µJ/mm^3^). These SED thresholds were used to analyze the best implant dimensions that would preserve the maximum buccal bone thickness while avoiding peri-implant bone resorption due to occlusal forces.

Figure 3(A,B) presented the peri-implant SED distribution for the eight simulated models using different color range adjustments. For the former, the scale was adjusted to present in gray the regions with SED ≥ ${Sp}_{c},$ and for the latter, the scale of each bone was adjusted to present in gray the regions with SED higher than their own Sp values (SED ≥ ${Sp}_{c}$ for cortical bone, and SED ≥ ${Sp}_{t}$ for trabecular bone). Figure 3(B) facilitates a more accurate interpretation of trabecular peri-implant bone resorption risk, highlighting the more vulnerable areas (gray and red regions) for both cortical and trabecular bones. On the contrary, the use of the same range for cortical and trabecular (Figure 3(A)) compromises the result interpretation and should be avoided, since it induces an underestimation of the trabecular bone failure. The same issue occurs when some authors convert the 4,000 µstrain limit into a stress limit [25]. The stress limit of the cortical bone is 10 times higher than the value of the trabecular bone (54.8 MPa and 5.48 MPa, respectively) when assuming the bone elastic modulus used in this study. This difference is usually ignored by some FEA studies.

In general, Figure 3 shows that the peri-implant bone SED decreased with the increase of implant diameter and length. The highest SED values could be seen at the cortical-trabecular interface of the buccal crest, close to the implant shoulder level, which has been already highlighted by other FE studies [30]. In the anterior maxilla zone, even minimal bone resorption at the buccal crest can promote a negative impact on the aesthetic outcome of the prosthetic treatment, due to the higher risk of peri-implant soft-tissue recessions. Although a color map of the FEA output distribution presents valuable information regarding failure risk, it is not easy to interpret, especially for a dentist that is not familiar with FEA.

To facilitate the clinical decision-making process regarding implant dimension selection, the cortical and trabecular top ten SED values of ROI for each model were used to calculate the peri-implant bone resorption risk index (Equations (5) and (6)). Figure 4 shows that model Ø4.00 presented medium trabecular PIBR_ri_ for L10 and low index for L13, indicating a relevant effect of increasing the implant length. Still, for cortical bones, both implant lengths presented low-risk indexes. When intermediate implant diameters were simulated, cortical bone showed low-risk indexes and trabecular bone showed medium ones. For models Ø3.25, high PIBRri were observed for both trabecular and cortical bones. The reduction of the bone-implant interface area increased the mechanical stimulus at peri-implant bones, driving both cortical and trabecular bones to the pathological overload window. For these models, the increase of 3 mm in length was not sufficient to compensate for the decrease in bone-implant interface area due to diameter reduction. Therefore, from a biomechanical point of view, there is no advantage to preserve the recommended 2 mm BBT by using a narrow implant of 3.25 mm.

A recent cohort study [13] compared aesthetic and functional outcomes between narrow and standard-diameter implants (NDI = 3.3 mm and SDI = 4.1 mm) in the anterior maxilla. The study evaluated 32 implant-supported single crowns in a follow-up period of 1 to 6 years. NDI revealed a higher Periotest value than SDI, which was associated with the smaller bone–implant interface. However, similar aesthetic assessment and patients’ satisfaction between groups and a 100% survival rate was observed. The authors highlighted that the high survival rate could be justified by the control protocols of the clinical study, which include a strict patient selection and an experienced surgeon team. Meanwhile, a systematic review [31] indicated that NDI with diameters between 3.0 mm and 3.25 mm showed a significantly lower survival rate when compared with implant diameters of 3.3 mm and 3.4 mm.

The current study was designed to simulate a unique geometry of an anterior maxillary alveolar process for all implant sizes. Therefore, as the implant diameter decreased, the buccal bone thickness increased (Table 1). Except for this aspect, the computational models enabled a complete control of research variables, which is an important advantage over clinical studies since the effect of the study variable is not adulterated by the effect of uncontrolled ones. However, the observed peri-implant bone resorption risk indexes are valid for a similar clinical scenario, with a favorable anatomy condition, in which bone augmentation is not necessary. In the clinical decision-making process, the dentist needs to evaluate other variables that influence the peri-implant strain and stress, such as intensity of masticatory forces [32], occlusal pattern, presence of parafunction, implant position and inclination [33], bone quality [34], crown-to-implant ratio, the distance between the implant neck plan and the load application point, and the heterogeneity of peri-implant bone properties.

The finite element models used in this study represented a maxillary D3 bone (type III), with a one-millimeter cortical layer surrounding a homogeneous trabecular bone. Even with the huge improvement in computer science in the last decades, it is still impractical to represent the microarchitecture of trabecular bone and the dental implant in the same model, due to the scale difference between the individual trabeculae (50 µm to 200 µm thickness) and the implant-crown unit (approximately 20 mm length). Therefore, in single-scale models, the porous trabecular bone is usually characterized by the averaged mechanical stiffness of the trabeculae and the marrow space [35,36]. Multiscale finite element analyses have been proposed to represent the complex and heterogeneous microstructure of bone in femur fracture risk studies [37,38], but this approach is still unusual in Dentistry. Nevertheless, with the increasing interest in patient-specific FE models and the continuous improvement of computer technology, it is foreseeable that multiscale FE models will replace the current strategy to predict bone mechanical failures based on clinical experience [38].

There are other limitations in the current FE models that must be considered when extrapolating the results to clinical situations, especially the assumption of a complete osteointegration (bone-implant interface was perfectly bonded) and the representation of a static loading condition. Additionally, the calculated indexes were restricted to peri-implant bone resorption risk due to occlusal overload after the osseointegration process. Resorption risks related to surgical protocol, peri-implantitis, or patients’ systemic condition that interferes with the bone remodeling mechanism were not considered.

## Conclusion

According to the clinical scenario simulated in this study, including the assumption of homogeneity for the trabecular bone, the preservation of a 2 mm buccal bone thickness by selecting a narrow implant of 3.25 mm should be avoided, since it generates high peri-implant bone resorption risk induced by occlusal overload.
